# Folate-Decorated Cross-Linked Cytochrome c Nanoparticles for Active Targeting of Non-Small Cell Lung Carcinoma (NSCLC)

**DOI:** 10.3390/pharmaceutics14030490

**Published:** 2022-02-24

**Authors:** Irivette Dominguez-Martinez, Freisa Joaquin-Ovalle, Yancy Ferrer-Acosta, Kai H. Griebenow

**Affiliations:** 1 Department of Chemistry, University of Puerto Rico, San Juan 00925, Puerto Rico; irivette.dominguez@upr.edu (I.D.-M.); freisa.joaquinovalle@upr.edu (F.J.-O.); 2 Molecular Sciences Research Center, San Juan 00926, Puerto Rico; 3 Department of Neuroscience, Universidad Central del Caribe, Bayamón 00956, Puerto Rico

**Keywords:** cancer, crosslinker, cytochrome c, drug delivery, folate receptor, Lewis lung carcinoma, triggered release

## Abstract

The folate receptor alpha (FR), which is overexpressed in solid tumors including NSCLC, can be utilized for active tumor targeting to afford more effective cancer therapies. In this context, cytochrome c (Cyt c) has drawn attention to cancer research because it is non-toxic, yet, when delivered to the cytoplasm of cancer cells, can kill them by inducing apoptosis. Cyt c nanoparticles (NPs, 169 ± 9 nm) were obtained by solvent precipitation with acetonitrile, and stabilized by reversible homo-bifunctional crosslinking to accomplish a Cyt-c-based drug delivery system that combines stimulus-responsive release and active targeting. Cyt c was released under intracellular redox conditions, due to an S–S bond in the NPs linker, while NPs remained intact without any release under extracellular conditions. The NP surface was decorated with a hydrophilic folic acid–polyethylene glycol (FA–PEG) polymer for active targeting. The FA-decorated NPs specifically recognized and killed cancer cells (IC_50_ = 47.46 µg/mL) that overexpressed FR, but showed no toxicity against FR-negative cells. Confocal microscopy confirmed the preferential uptake and apoptosis induction of our NPs by FR-positive cancer cells. In vivo experiments using a Lewis lung carcinoma (LLC) mouse model showed visible NP accumulation within the tumor and inhibited the growth of LLC tumors.

## 1. Introduction

Lung cancer is the leading cause of worldwide cancer deaths. Non-small cell lung carcinoma (NSCLC) is the most common type of lung cancer, accounting for 85% of the reported cases, and is associated with poor prognosis—a five-year survival rate of only 15% [1,2]. Although the current first-line anticancer agents (e.g., cisplatin and Taxol^®^) against NSCLC have been successful to some extent, their main drawbacks are their non-specific targeting, high dose requirements, poor bioavailability, the development of multiple drug resistance, and adverse side effects [3,4,5]. For example, NSCLC patients treated with cisplatin often suffer severe nephrotoxicity [6]. In principle, these effects arise from the chemotherapeutic agents’ lack of tumor selectivity and systemic toxicity without discriminating healthy tissues, producing unwanted and often severe and dangerous side effects.

All chemotherapeutic drugs, regardless of their specific target or mechanism of action, produce the same cytotoxic end effect in sensitive cells: cell death. However, the apoptotic DNA damage response requires the involvement of the p53 tumor suppressor pathway, which is mutated/inactivated in ~50% of human cancers [7] and approximately 70% of lung adenocarcinoma cases [8]. Such oncogenic mutations that disrupt the apoptosis pathway contribute to tumor initiation, progression, and metastasis [9]. For example, Taxol^®^ causes damage leading to p53-tumor-suppressor-dependent apoptosis and often results in the development of resistance, leading to therapy failure and relapse [4]. Such limitations of conventional chemical drugs have spurred efforts to identify more effective chemotherapeutic agents that can be tolerable in higher doses and act independently of the p53 pathway [10].

As an alternative approach, proteins that exhibit potent cytotoxic activities can be exploited to develop new anti-tumor drugs [11]. Cytochrome C (Cyt c) fulfills this requirement because it is non-toxic in the cytoplasm and acts downstream in the apoptosis cascade, thus evading many steps with potential mutations. During Cyt c-mediated apoptosis, the apoptosome formation (Apaf-1/Cyt c complex), which cleaves procaspase-9 to active caspase 9, is a critical event responsible for activating effector caspases 3 and 7, which mediate apoptosis [12,13]. Indeed, using a drug delivery system to transport Cyt c into the cytoplasm of cancer cells could help overcome any failure in activating the intrinsic or ‘mitochondrial’ apoptotic signaling pathway that prevents Cyt c release [10,14,15]. Hence, Cyt c has drawn the attention of groups in the field for its potential to be developed into a highly effective and selective anticancer drug [13]. However, since Cyt c is a cell-membrane-impermeable protein, it must be linked to an uptake process.

Folic acid (FA) is a B vitamin necessary for cellular proliferation and DNA synthesis and modification [16]. The folate receptor alpha (FR) is a well-known tumor marker that is overexpressed in 40% of human cancers, and it is rarely expressed or inaccessible in most normal cells [17]. Studies have found that levels of FR expression are associated with tumor stage and survival, specifically in lung adenocarcinomas [18,19]. This overexpression in tumors promotes folic acid ligand–drug conjugates to bind and promote their subsequent uptake via receptor-mediated endocytosis [20]. Hence, FA has been extensively used as a ligand to improve tumor therapy’s uptake and target cancerous cells.

One of the most influential hallmarks of cancer cells is their ability to sustain proliferative and pro-angiogenic signaling, which leads to an unstable and leaky vasculature accompanied by insufficient lymphatic drainage in tumors (the EPR effect), which drives the accumulation of nano-sized delivery systems in solid tumors [21]. The EPR effect alone increases the tumor specificity of nanoparticles (NPs) by 20–30% over critical normal organs [22]. The polyethylene glycol (PEG) polymer has been used to modify NPs and overcome their low stability, immunogenicity, and short blood circulation half-life [23]. Combining the passive EPR-mediated targeting with an additional tumor-abundant ligand such as FA not only amplifies the specificity of therapeutic NPs, but also facilitates their cellular uptake.

For in vivo applications, shape and size are critical determinants of nanoparticle uptake and circulation [24,25]. Spherical particles that are 100–200 nm in size have the highest potential for prolonged circulation, because they are large enough to avoid uptake in the liver (particles over 300 nm accumulate in the liver) but small enough to prevent filtration to the spleen (as the spleen has fenestrations that do not exceed 200–500 nm) [26]. Typically, nanoparticles are trapped by mechanical filtration in the spleen sinusoids, followed by removal from circulation by the cells of the reticuloendothelial system in the liver [23].

To date, various nano vehicles have been explored to facilitate the intracellular delivery of Cyt c for therapeutic purposes with different degrees of success [27,28]. Recently, our research group overcame biocompatibility and off-target limitations commonly seen in anticancer therapeutics by designing a Cyt c-based drug delivery system (DDS) coated with a biodegradable polymer, PLGA–PEG–FA, which is 253 nm in size [29]. This DDS showed a tumor-targeting capability and no cytotoxicity after an in vivo injection using a lung carcinoma immune-competent mouse model [29].

Herein we substantially simplify the system by employing another strategy for preventing protein dissolution in buffer and blood, which uses redox-sensitive crosslinking. Cyt c NPs were prepared by solvent precipitation. Protein nanoprecipitation is an easy and reproducible technique to prepare Cyt c NPs. Their size, shape, and surface charge can be controlled to incorporate passive, active, and stimuli-responsive targeting [30]. Next, the Cyt c-based NPs were stabilized by homo-bifunctional reversible crosslinking using dithiobis(succinimidyl propionate) (DSP). This crosslinker contains a disulfide bond that is reduced under intra-cellular conditions, thus affording the dissolution of the NPs in the cytoplasm of target cells. To achieve receptor-mediated internalization by FR-overexpressing cancer cells, we conjugated folate-poly(ethylene glycol) (FA–PEG) to the surface of the NPs. Our data demonstrate a substantial improvement over our previous Cyt c delivery system both in vitro, using the Lewis lung carcinoma (LLC) cell line, and in vivo, using the LLC mouse model. This mouse model is a practical in vivo approach to study drug safety and test whether targeted NP therapies reach their target in the presence of a functional immune system [31].

## 2. Materials and Methods

### 2.1. Materials

Cyt c from the equine heart (≥95% purity), acetonitrile, dithiobis(succinimidyl propionate) (DSP) crosslinker, L-glutathione (reduced, ≥98.0% purity), and isomer I of fluorescein isothiocyanate (FITC) were obtained from Sigma-Aldrich (St. Louis, MO, USA). Folate–poly(ethylene glycol)–succinimidyl ester (FA–PEG–NHS, M_W_ 3400 Da) was purchased from Biochempeg Scientific Inc. (Watertown, MA, USA). CellTiter 96 aqueous non-radioactive cell proliferation assay was purchased from Promega Corporation (Madison, WI, USA). Ampilite^TM^ Colorimetric Caspase 3/7 Assay Kit was purchased from AAT Bioquest (Sunnyvale, CA, USA). DAPI (4′,6-diamidino-2-phenylindole, NucBlue^®^), FM-464 membrane stain, propidium iodide (PI), and CellEvent^TM^ Caspase-3/7 Green was obtained from Invitrogen (Eugene, OR, USA). Near-infrared reactive dye IRDye^®^ 680RD was available as a protein labeling kit (high molecular weight) from LI-COR Biosciences.

### 2.2. Synthesis of Cross-Linked Cyt c–PEG–FA Nanoparticles

Crosslinked Cyt c–PEG–FA NPs were synthesized by first obtaining protein NPs using a nanoprecipitation method [29]. Briefly, 5 mg/mL of Cyt c dissolved in ultrapure water was solvent-precipitated by adding acetonitrile at a 1:4 (water/acetonitrile) volume ratio at a constant rate of 300 mL/h using an automated syringe pump. The NP suspension was left stirring for 5 min. Subsequently, the resulting Cyt c NP suspension was covalently stabilized by directly adding 0.2 mg/mL of the homo-bifunctional DSP crosslinker dissolved in acetonitrile. After 30 min under constant stirring at room temperature, 7 mg/mL of FA–PEG–NHS (MW 3400 Da) polymer dissolved in a mixture of 3:1 acetonitrile/ultrapure water was added to the NP suspension and was allowed to react at room temperature for 18 h. The NPs were subsequently centrifugated at 10,000 rpm and washed thrice with ultrapure water. These NPs were then flash-frozen and freeze-dried.

### 2.3. Determination of Precipitation Efficiency and Actual Protein Loading

To calculate precipitation efficiency and actual drug loading, an aliquot of 10 μL was collected right before nanoprecipitation to determine the initial amount of Cyt c. After nanoprecipitation, the NP suspension was centrifuged for 10 min at 10,000 rpm at room temperature. The concentrations of Cyt c in the aliquot and supernatants were determined by measuring the absorbance at 410 nm using a NanoDrop 2000c (Thermo Scientific, Waltham, MA, USA) [32]. The final amount of NP was obtained by weighing the final product. Precipitation efficiency (EE) and actual protein loading (AL) were calculated using the following equations:

(1) $$\mathrm{EE}\;\left(\%\right)=\frac{\mathrm{initial}\;\mathrm{amount}\;\mathrm{of}\;\mathrm{Cyt}\;c-\;\mathrm{Cyt}\;c\;\mathrm{in}\;\mathrm{supernatant}}{\mathrm{initial}\;\mathrm{amount}\;\mathrm{of}\;\mathrm{Cyt}\;c}\times 100$$

(2) $$\mathrm{AL}\;\left(\%\right)=\frac{\mathrm{mg}\;\mathrm{of}\;\mathrm{Cyt}\;c\;\mathrm{in}\;\mathrm{nanoparticles}}{\mathrm{mg}\;\mathrm{of}\;\mathrm{nanoparticles}}\times 100$$

The experiments were performed in triplicate, averaged, and standard deviations (SD) were calculated.

### 2.4. Dynamic Light Scattering (DLS)

Particle size, polydispersity index (PDI), and zeta potential of Cyt c NPs, crosslinked Cyt c NPs, and crosslinked Cyt c–PEG–FA NPs were determined by dynamic light scattering (DLS) using a Zetasizer Nano ZS (Malvern Panalytical Ltd., Malvern, UK). The samples were dispersed in ultrapure water and subjected to ultrasonication at 240 W for 30 s before the measurements. NPs were transferred to capillary cells for zeta potential determination. The experiments were performed in triplicate, and the results were expressed as the mean ± SD.

### 2.5. Scanning Electron Microscopy (SEM)

SEM micrographs of crosslinked Cyt c–PEG–FA NPs were performed using a JEOL 6480LV scanning electron microscope at 20 kV. Lyophilized NPs were coated with gold for 10 s using an auto sputter coater (108 Auto/SE, Ted Pella Inc., Redding, CA, USA).

### 2.6. In Vitro Release

To determine the in vitro Cyt c release profile of NPs, 0.5 mg/mL of crosslinked Cyt c–PEG–FA NPs were suspended in PBS buffer (pH 7.4) with glutathione (GSH) concentrations of 0, 0.001, and 10 mM simulating extra- and intracellular conditions [33]. The NPs were incubated at 37 °C under constant stirring for various time intervals: 0.5, 2.5, 20, 30, and 46 h. At predetermined time points, NPs were centrifuged at 14,000 rpm for 10 min, and the supernatant was collected and replaced with an equal volume of PBS/GSH buffer. The supernatant was used to determine the concentration of released Cyt c by UV-vis spectroscopy using a NanoDrop 2000c (Thermo Scientific, Waltham, MA, USA). The wavelength used to measure the concentration of non-reduced Cyt c (0 mM and 0.001 mM GSH) was 530 nm, and reduced Cyt c (10 mM GSH) was 550 nm [29]. The amount of released Cyt c from the NPs to the PBS/GSH dissolvent (observed at 550 nm) was used to construct cumulative Cyt c release profiles at incrementally higher reducing conditions using GSH as the reducing agent. The experiments were performed in triplicate, the results averaged, and the standard deviations calculated.

### 2.7. Cell-Free Caspase 3/7 Activity Assay

Caspase activation by Cyt c was measured in LLC cell lysate following the procedure previously reported in the literature [29]. Briefly, 5 × 10^6^ LLC cells were resuspended in 100 µL of lysis buffer, and cells were lysed with four freeze–thaw cycles using liquid nitrogen and a water bath at 37 °C. Then, the cell lysate was centrifuged at 11,000 rpm for 20 min at 4 °C, and the supernatant (lysate) was collected. For the caspase 3/7 cell-free reaction, the obtained lysate was mixed with 300 µg/mL of crosslinked Cyt c–PEG–FA NPs using a volume ratio of 1:1 (lysate/NPs). The reaction was incubated at 37 °C for 150 min. We used native Cyt c and untreated cells (lysate only) as a control experiment under the same conditions. Afterward, the Caspase 3/7 assay was performed following the manufacturer’s protocol (Ampilite^TM^ Colorimetric Caspase 3/7 Assay; AAT Bioquest, Sunnyvale, CA, USA). In a 96-well plate, 100 µL of the active lysate was mixed with 100 µL of the Caspase 3/7 working reagent. Then, the plate was incubated at room temperature for 1 h, and the absorbance was measured at 490 nm using a Synergy H1 (BioTek, Winooski, VT, USA). The mean ± SD of the cell-free caspase 3/7 activity was obtained from two independent experiments performed in triplicate. The results were analyzed statistically using the unpaired Student’s *t*-test by GraphPad Prism 9.1.1 (**** *p* < 0.0001, *n* = 6).

### 2.8. Cell Viability Assay

MTS cell viability assay (CellTiter 96 aqueous non-radioactive assay) from Promega (Madison, WI, USA) was used to measure the half-maximal inhibitory concentration (IC_50_) value for the crosslinked Cyt c–PEG–FA NPs in LLC cancer cells. Lewis lung carcinoma (LLC) cells (10,000 cells/well) were seeded in a 96-well plate and incubated with serial dilutions (300, 200, 100, 50, 25, and 12.5 μg/mL) of crosslinked Cyt c–PEG–FA NPs for 24 h at 37 °C. Controls, such as 300 μg/mL native Cyt c, FA–PEG-NHS, and folate-free Cyt c–DSP NPs, were also tested. As a control experiment, FR-negative mouse embryonic fibroblasts (NIH/3T3) cells and FR-positive human cervical carcinoma (HeLa) cells (10,000 cells/well) were also incubated with 300 μg/mL of crosslinked Cyt c–PEG–FA NPs for 24 h. MTS assay was performed following instructions from the kit manufacturer, and the absorbance was measured at 490 nm using a microplate reader (Tecan Infinite 200 Pro, Meilen, Zurich, Switzerland). The IC_50_ value was calculated using GraphPad Prism from the dose-response curve: X = log(X) against the normalized Y (values being 0% for the smallest value in the data set and 100% for the highest value data set). The normalized percent of cell viability was plotted against the following log concentrations of crosslinked Cyt c–PEG–FA NPs after 24 h: 1.097, 1.398, 1.699, 2.00, 2.301, and 2.477 μM. Results were expressed as mean values of independent experiments performed in triplicate (*n* = 9) ± SD. To test the ability of folate to help reduce cancer cell viability, we compared folate-targeted Cyt c NPs and folate-free Cyt c NPs MTS results using an unpaired Student’s *t*-test analysis (GraphPad Prism 9.1.1). A difference between folate-targeted Cyt c NPs and folate-free Cyt c NPs cell viability at 300 μg/mL was found, resulting in a statistically significant difference with a **** *p*-value of <0.0001.

### 2.9. In Vitro Cellular Internalization and Endosomal Escape of Cross-Linked Cyt c–PEG–FA NPs

The crosslinked Cyt c–PEG–FA NP cellular internalization and release into the cytoplasm was observed by confocal laser scanning microscopy (CLSM) in vitro. LLC and NIH/3T3 cells (10,000 cells/well) were seeded in chambered cover glass plates (with 4 wells). For these experiments, crosslinked Cyt c–PEG–FA NPs were modified by attachment of a fluorescein isothiocyanate (FITC) molecule (495 nm excitation wavelength) in the protein’s amino group. Briefly, 25 μL FITC (1 mg/mL) was added to 1 mL of NP sample (3 mg/mL) dissolved in PBS (pH 7.4) buffer. The reaction of NPs and FITC was stirred at 4 °C overnight (~16 h) and covered to block out light and minimize fluorophore quenching. The FITC-labeled NPs were further lyophilized and dissolved in cell culture media at the time of use.

Cell lines were grown at 37 °C in 5% CO_2_ for 24 h with both: FITC-labeled NPs (100 μg/mL) and the endosome marker FM-464 (10 μg/mL). Afterward, the medium was removed, and the cells were washed with PBS three times, followed by fixation with 3.7% formaldehyde. After fixation, cells were incubated with DNA fluorescent stain DAPI (358 nm excitation wavelength) at 1 μg/mL in PBS for 5 min, followed by three washes with PBS alone. For cell imaging, 90% glycerol in PBS was used as mounting medium. Untreated cells were used as control. For cellular internalization and endosomal escape analysis, the chambered cover glass plates were examined under a Nikon Eclipse Ti-E inverted confocal scanning microscope (Nikon Instruments Inc., Melville, NY, USA) using a 40× oil immersion objective and excitation at 488 nm. Quantification of the FITC fluorescence intensity of NPs in the red-stained cell membrane area was determined using the NIS-Element AR analysis program. The difference between the intensities of NP-treated cells, and untreated cells (*p* < 0.05) was used to subtract the green background autofluorescence. Unpaired *t*-test analyses were performed using GraphPad Prism software.

### 2.10. Study of Cell Death Induction by Confocal Laser Scanning Microscopy (CLSM)

LLC cells and NIH/3T3 cells (10,000 cells/well) were seeded on 4-well chambered cover glass plates. The cells were treated with 100 μg/mL of cross-liked Cyt c–PEG–FA NPs at 37 °C for 24 h. To detect apoptosis and nuclear fragmentation in cells, these were washed with PBS (1×) and incubated with the apoptotic cell marker propidium iodide (75 μM) for 5 min. Cells were fixed with 3.7% formaldehyde and incubated with DAPI nuclear stain, followed by three cycles of PBS washing. As a mounting medium, 90% glycerol in PBS was used. The chambered cover glass plates were examined under a Nikon Eclipse Ti-E inverted confocal scanning microscope (Nikon Instruments Inc., Melville, NY, USA) using a 40× oil immersion objective. Untreated cells were subjected to DAPI/PI incubation and used as a control.

### 2.11. Study of the Apoptotic Induction Mechanism by Caspase 3/7 Green Detection

LLC cells (10,000 cells/well) were seeded in 4-well chambered cover glass plates. The cells were incubated at 37 °C for 24 h with 100 μg/mL of cross-liked Cyt c–PEG–FA NPs. The activity of caspase 3/7 was determined with the CellEvent^TM^ caspase 3/7 green reagent (Invitrogen) according to the manufacturer’s instructions. The chambered cover glass plates were examined under a Nikon Eclipse Ti-E inverted confocal scanning microscope (Nikon Instruments Inc., Melville, NY, USA) using a 40× oil immersion objective. Untreated cells were subjected to DAPI/CellEvent^TM^ incubation as well and used as a control. The mean green intensity of the confocal images was measured using the NIS-Element AR analysis program. Unpaired *t*-test analysis by GraphPad Prism comparing NPs-treated cells and untreated cells was considered statistically significant within the 95% confidence interval (*p* < 0.05).

### 2.12. Studies to Detect NPs Organ Distribution

Our in vivo studies had two different purposes: (1) to test the in vivo tumor-targeting capacity of our new NP system, and (2) to test the efficacy of the NPs in tumor growth. We answered the first question following our previous methodology from Barcelo-Bovea et al. (2020), where we observed NP binding through the different organs at different time points. However, the strongest infrared signal was 5 min after intravenous injection and was almost undetectable after 1 h. Studies have shown that folate receptor uptake can be as fast as 30 min or less in cancer cells [34]. Because the diameter of the studied NPs was ~169 nm, which is 85 nm smaller than our previous prototypes, we expected their kinetics to be faster and used the short-time observation window of 5 min to determine whether the nanoparticles reached the tumor target. We chose the intravenous route for these experiments because there is no absorption limitation ‘barrier’; it has a fast onset of action, and is clinically applicable. Nevertheless, it is a challenging technique, especially for repeated administrations, and it has a limited volume application (less than 100 μL in mice).

To test tumor and organ targeting by NPs after entering the blood circulation, we used a syngeneic (immune-competent) C57BL6J mouse model called Lewis lung carcinoma (LLC). We used 14-week-old mice (C57BL6J wild type strain, male, from Jackson Labs, Bar Harbor, ME, USA) of 25 g in weight. To develop the Lewis lung carcinoma in these mice, we first grew the LLC murine lung carcinoma cells (commercially obtained, derived from a C57BL6J mouse lung tumor) in a flask, and, after confluency, cells were quantified and injected subcutaneously. LLC mouse cancer cells were cultured in high-glucose Dulbecco’s modified Eagle’s medium (DMEM) supplemented with 10% fetal bovine serum (FBS) and 1% penicillin/streptomycin/amphotericin (PSA) to confluency. Cells were gently removed from the culture flask using a cell scraper, centrifuged at 1500× *g* (5 min), and quantified. After quantification, 1 × 10^7^ LLC cells were obtained and added to a 1.5 mL tube to a total volume of 200 μL with cell media. This 200 μL of 1 × 10^7^ cells was added to 200 μL of extracellular matrix (ECM) growth factor reduced gel from Engelbreth-Holm-Swarm murine sarcoma (Sigma-Aldrich, St. Louis, MO, USA), and they were gently mixed by pipetting. This total of 400 μL cells plus ECM was subcutaneously injected into the upper-right dorsal area of the mouse body using a 26 gauge needle to induce tumor growth.

Mouse tumors were grown up to 15 d after implant. These mice were injected by tail vein route with infrared-labeled NPs (IRDye 680RD-labeled NPs) to visualize these. An amount of 0.15 mg of NPs was administered in a volume of 200 μL, following the protocol of Barcelo-Bovea et al. (2020) [30]. Five minutes after tail vein injection, mice were subsequently euthanized. Tumor and organs (brain, heart, lungs, spleen, kidneys, liver, and intestines) were quickly extracted and scanned for IR-labeled NP distribution using the LI-COR Odyssey CLx infrared scanner. For the tumors, a 42 μm resolution and high-quality setting were chosen in Image Studio^TM^ software (Lincoln, NE, USA). For the organs, a 337 μm resolution was used. All the scanned tissue area in the 2-dimensional image was selected for quantification, and the IR signal was scanned and analyzed using Image Studio^TM^. The percentage increase in infrared signal from the control mouse at 5 min after tail vein injection was calculated following Barcelo-Bovea et al. (2020).

All necessary approvals from the Institutional Animal Care and Use Committee (IACUC) were in place for the performed research: Assurance ID number D16-00343; IACUC Protocol Universal Number 048-2021-08-01-PHA-IBC.

### 2.13. Studies to Determine NPs Tumor Decrease in Mice

Given the efficiency of NPs targeting the tumor tissue, we tested their efficiency in decreasing tumors in mice using the Lewis lung carcinoma (LLC) mouse model. Adult mice ranging from 36 to 60 weeks old, representing an age from adulthood to the reproductive senescence period, were selected for our studies. These mice were implanted with cells to grow a tumor for a total of 12 days. Mice were injected intraperitoneally with 7 mg/kg Cyt c nanoparticles of 169 nm at day 3, as an early-tumor stage intervention, and at day 8, as a late-stage intervention in tumor growth.

Tumor volume was measured manually by caliper every 3 days using the length and width of the tumors:

(3) $$\mathrm{Tumor}\;\mathrm{volume}=\frac{\mathrm{Tumor}\;\mathrm{length}\times {\left(\mathrm{Tumor}\;\mathrm{weigth}\right)}^{2}}{2}$$

The percentage of tumor growth from day 3 and day 12 (last day) was calculated using the following formula:

(4) $$\mathrm{Tumor}\;\mathrm{growth}=\left(\frac{\mathrm{Volume}\;\mathrm{at}\;\mathrm{day}\;12-\mathrm{Volume}\;\mathrm{at}\;\mathrm{day}\;3}{\mathrm{Volume}\;\mathrm{at}\;\mathrm{day}\;12}\right)\times 100$$

A total of *n* = 6 mice were untreated, and *n* = 7 mice were treated with NPs. An unpaired *t*-test with a Kolmogorov–Smirnov analysis by GraphPad Prism between NP-treated and untreated mice was considered statistically significant within the 95% confidence interval at *p* = 0.0385. Animal weight was also measured to monitor drug safety, as a sharp decrease in body weight (more than 15–20% during the experiment) is considered unhealthy in tumor models [35]. No difference in mouse weight between groups was observed.

## 3. Results

### 3.1. Synthesis and Characterization of Cross-Linked Cyt c–PEG–FA Nanoparticles

After nanoprecipitation, the diameter of the crosslinked Cyt c NPs was 164 ± 5 nm, and Cyt c–PEG–FA NPs was 169 ± 9 nm (Table 1) as determined by dynamic light scattering (DLS). The NPs generated here were smaller compared to the core-shell Cyt c delivery systems previously developed by our group. The decrease in the zeta potential of the crosslinked Cyt c–PEG–FA NPs (17.7 ± 1.7 mV) compared with the Cyt c–DSP NPs (27.5 ± 3.9 mV) confirmed the successful attachment of the FA–PEG–NHS targeting polymer to the amino groups of Cyt c, leading to a reduction in the number of positive charges on the NPs’ surfaces.

**Figure 1:**
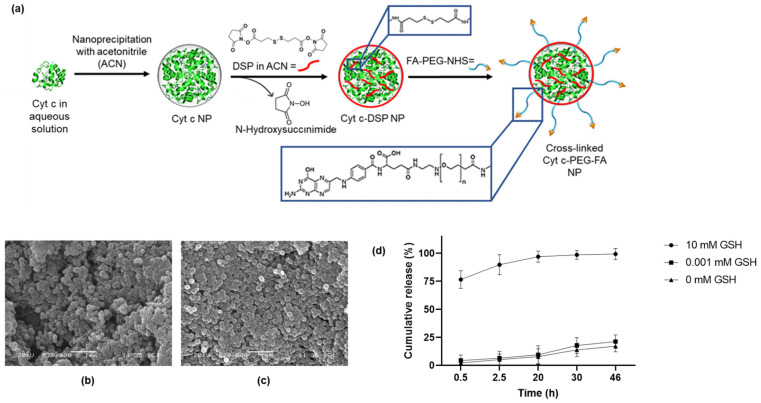
(**a**) Synthesis route of crosslinked Cyt c–PEG–FA NPs. SEM micrographs of (**b**) crosslinked Cyt c NPs and (**c**) crosslinked Cyt c–PEG–FA NPs. (**d**) Cumulative in vitro release profile of Cyt c from crosslinked Cyt c–PEG–FA NPs at 37 °C. NPs were dissolved in PBS buffer with zero GSH (triangles), 0.001 mM GSH (squares), and 10 mM GSH (circles) to simulate extracellular (non-reducing) and intracellular (reducing) physiological conditions. Data are the mean ± SD of experiments performed in triplicate. Statistical analysis by ordinary one-way ANOVA multiple comparison analysis demonstrated a significant difference between the intracellular and extracellular conditions when compared with the control, *p* < 0.0001.

Scanning electron microscopy (SEM) was performed to examine the shape of the NPs (Figure 1b,c). The SEM images of lyophilized crosslinked Cyt c NPs and crosslinked Cyt c–PEG–FA NPs showed a spherical shape with a narrow size distribution.

### 3.2. In Vitro Redox-Responsive Release

To investigate the cumulative release profile of the crosslinked Cyt c–PEG–FA NPs, we used 10 mM glutathione (GSH) to simulate intracellular conditions, 0.001 mM GSH to simulate extracellular conditions, and 0 mM GSH as a control [36]. The cumulative Cyt c release profile is shown in Figure 1d. The crosslinked Cyt c–PEG–FA NPs exhibited an efficient release profile under reducing conditions (10 mM GSH); the most Cyt c was released in the first 0.5 h as a ‘burst release’. In contrast, we found that only about 17% and 21% of Cyt c were released using no or 0.001 mM GSH, respectively, and this release was slower than that with 10 mM GSH.

### 3.3. Cell-Free Caspase 3 and 7 Assay

**Figure 2:**
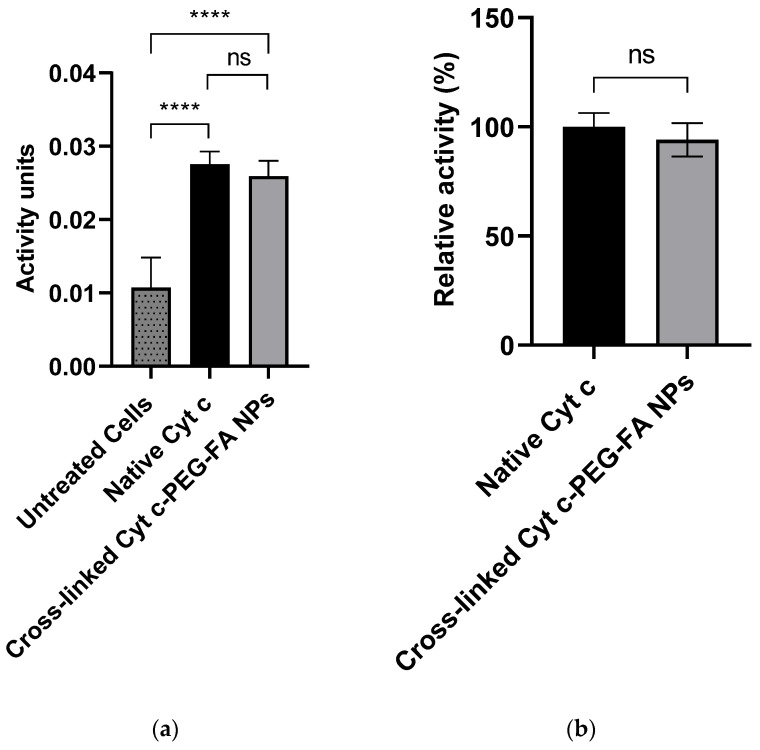
Activation of caspase 3/7 using a cell-free caspase assay. (**a**) Compared to untreated cells (control), crosslinked Cyt c–PEG–FA NPs activated caspase 3/7 to a significantly greater extent, similar to the activity provided by the native Cyt c protein. (**b**) Caspase 3/7 activation of crosslinked Cyt c–PEG–FA NPs compared with the caspase 3/7 activation by native Cyt c. LLC lysate treated with crosslinked Cyt c–PEG–FA NPs was able to activate the caspase 3/7 significantly, similar to that afforded by native Cyt c. The relative caspase activity offered by crosslinked Cyt c–PEG–FA NPs was not significantly different compared to the native Cyt c protein. **** Indicates a significant difference (*p* < 0.0001) in an unpaired *t*-test analysis with *n* = 6. Error bars represent the calculated SD.

We determined the potential of Cyt c to interact with Apaf-1 and induce apoptosis after the NP formulation. For this purpose, in vitro cell-free caspase 3/7 activity assays were conducted in a cell-free system (LLC lysate), and native Cyt c was used as a positive control. As shown in Figure 2, the addition of crosslinked Cyt c–PEG–FA NPs to fresh cytosol produced caspase 3/7 activation. Compared to the untreated cells, activation of caspase 3/7 was statistically significantly higher in cells treated with the crosslinked Cyt c–PEG–FA NPs at a *p*-value of <0.05 (Figure 2a). Cyt c–PEG–FA NPs had 94 ± 8% of their caspase activation activity with no significant difference compared to native Cyt c (Figure 2b). Therefore, since our NPs retained all of their enzyme activity, we demonstrated that the conjugation of the Cyt c NPs does not produce any adverse impact on the capability of the protein to induce apoptosis.

### 3.4. Cell Viability Assays

**Figure 3:**
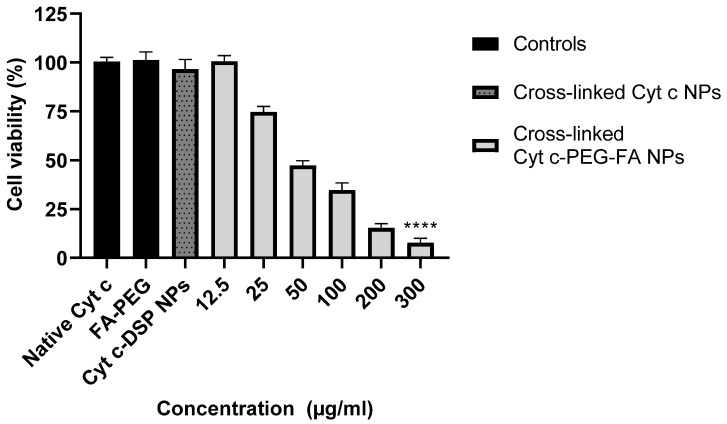
MTS cell viability assay of LLC cells after 24 h of incubation with folate-containing crosslinked Cyt c–PEG–FA NPs in a concentration-dependent manner. The percent of cell viability for the Cyt c–PEG–FA NPs is shown in gray columns, at increasing concentrations from 12.5 to 300 μg/mL. This dose-response curve was used to determine the IC_50_ value of the crosslinked Cyt c–PEG–FA NPs. As controls, we used the native Cyt c (protein alone, no NPs; first black column), PEG–FA (Folate–poly(ethylene glycol)–succinimidyl ester alone; second black column), and Cyt c–DSP NPs (Cyt c with the homo-bifunctional crosslinker DSP, without folate; gray dotted column). All controls were added at a concentration of (300 μg/mL). The cytotoxicities of the folate-free formulation and the folate-bearing NPs at the highest concentration were compared by unpaired *t*-test analysis (**** *p* < 0.0001, *n* = 9). Data shown are expressed as the mean ± SD.

The cytotoxicity evaluation of crosslinked Cyt c–PEG–FA NPs was performed using an MTS assay. A dose-response graph was constructed upon incubation of FR-positive LLC cells at different concentrations (12.5, 25, 50, 100, 200, and 300 µg/mL) of crosslinked Cyt c–PEG–FA NPs for 24 h. As shown in Figure 3, crosslinked Cyt c–PEG–FA NPs induced a significant reduction in the LLC cell viability in a dose-dependent manner after 24 h of incubation compared to untreated cells. The calculated IC_50_ value of the crosslinked Cyt c–PEG–FA NPs was 47.5 µg/mL (R^2^ = 0.9681). Additionally, the cell viability decreased at increasing concentrations of our NPs. These results demonstrate a clear correlation between the dose concentration of the NPs and its cytotoxic effect. As controls, LLC cells were incubated with native Cyt c, FA–PEG, and Cyt c–DSP NPs (folate-free, the crosslinker alone) at the highest NP concentration (300 µg/mL), as in the corresponding experiment, for 24 h. No significant cytotoxicity was observed after 24 h with either control compound. For the native Cyt c control, it was expected not to significantly affect the cell viability, since Cyt c is a cell-membrane-impermeable protein. As hypothesized, under the same conditions, folate-conjugated Cyt c NPs significantly reduced the cell viability of folate-receptor-expressing LLC cells compared to the folate-free Cyt c NPs (**** *p* < 0.0001, *n* = 9). These results validated the hypothesis that folate-conjugation was required for our NPs to address targetability and induce a cytotoxic potential.

**Figure 4:**
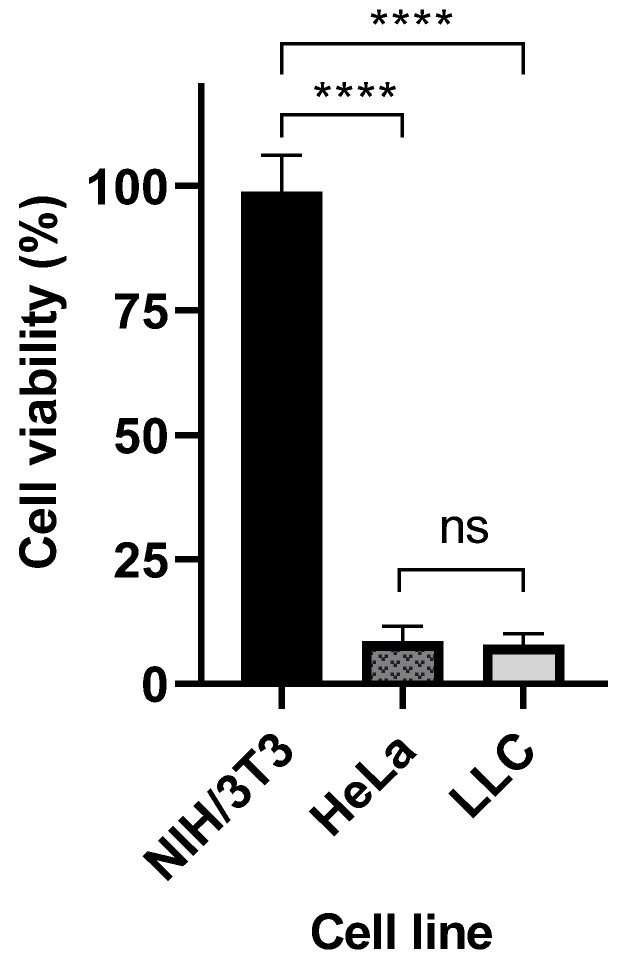
Comparison of the cytotoxicities of crosslinked Cyt c–PEG–FA NPs in cancerous and non-cancerous cell lines. Cell viability MTS assay after 24 h of NPs treatment at 100 µg/mL using FR-positive cells (LLC and HeLa cells) and FR-negative cells (NIH/3T3 cells). The mean ± SD was obtained from three independent experiments performed in triplicate. The results were analyzed statistically using an unpaired *t*-test (****, *p* < 0.0001, *n* = 9).

To evaluate the selective cytotoxic effect of crosslinked Cyt c–PEG–FA NPs for FR-positive cancer cells, we performed a comparative cell viability study with both FR-positive cells (the human cervical carcinoma HeLa cell line and LLC) and FR-negative cells (the mouse embryo fibroblasts NIH-3T3 cell line) [37,38]. As seen in Figure 4, crosslinked Cyt c–PEG–FA NPs significantly reduced the cell viability in FR-positive cell lines (LLC and HeLa cells) without a statistically significant difference (*p* < 0.05, *n* = 9). Furthermore, the viability of the NIH/3T3 cells was not significantly affected after 24 h of incubation with the folate-conjugated NPs. These results confirm that our FR-targeted DDS had a significant cytotoxic effect on cancer cells overexpressing FR but not on FR-negative, non-cancerous cells.

### 3.5. Study of Apoptotic Mechanism of Cell Death Induction by CLSM

**Figure 5:**
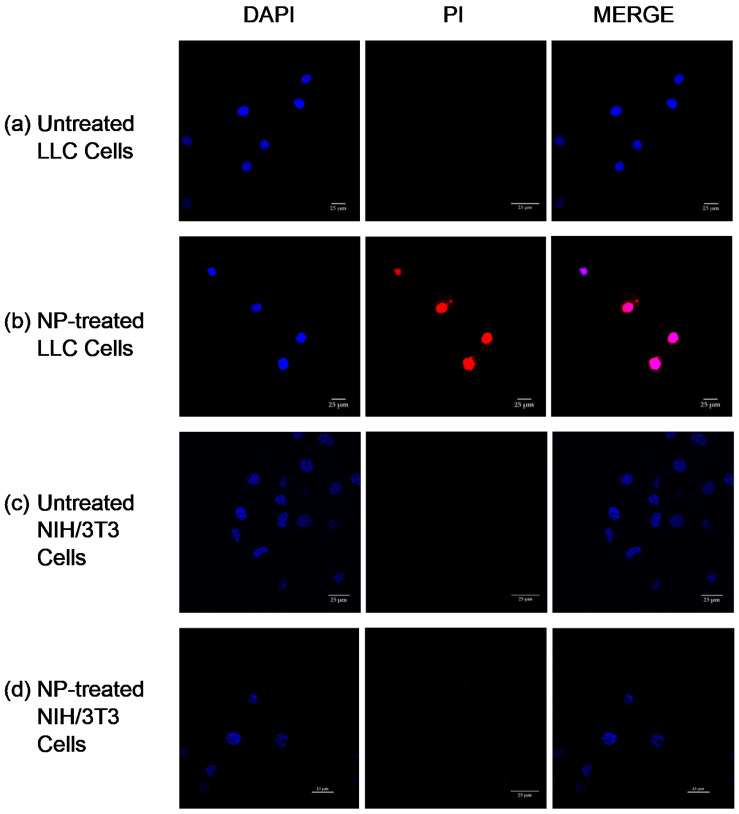
Study of DAPI and propidium iodide (PI) colocalization for the detection of apoptotic cells after 24 h of incubation with crosslinked Cyt c–PEG–FA NPs. (**b**) Selective induction of apoptosis was observed in LLC cells incubated with NPs. (**c**) No cellular apoptosis was observed in NIH/3T3 cells when incubated with NPs. (**a**,**d**) Untreated LLC and NIH/3T3 cells were used as controls, respectively.

To confirm whether the cell death was caused by apoptosis, we qualitatively evaluated the occurrence of nuclear segmentation, chromatin condensation, and PI presence in the cell nuclei after their treatment with NPs. LLC cells were treated with crosslinked Cyt c–PEG–FA NPs adjusted to a drug concentration of 100 μg/mL. After 24 h of incubation, the colocalization of DAPI and PI was determined by CLSM. In apoptotic cells, both dyes, PI (red) and DAPI (blue) localized in the nucleus due to the presence of pores in the cell membrane. The nuclei of the permeable LLC apoptotic cells were seen as a bright purple spot in the confocal images due to the colocalization of PI and DAPI (Figure 5). PI internalization is representative of highly condensed and fragmented chromatin in apoptotic cells. Thus, the red fluorescence of PI observed in the nucleus of LLC cells with crosslinked Cyt c–PEG–FA NPs confirmed that apoptosis cell death was occurring. In contrast, untreated LLC cells presented no indication of apoptosis, as could be observed by the lack of PI internalization due to the absence of red fluorescence in the confocal images. To evaluate the selectivity of our folate-decorated NPs, we compared the apoptotic cell death of FR-negative NIH/3T3 cells under the same conditions. Confocal images showed a lack of intense PI red fluorescence in the nuclei of NIH/3T3 cells treated with crosslinked Cyt c–PEG–FA NPs, whereas an enhanced red fluorescence in LLC cells was observed. These results are consistent with the intracellular assay and in vitro cell viability results described above, indicating that crosslinked Cyt c–PEG–FA NPs had a significant cytotoxic effect on cancer cells overexpressing FR but not on normal cells.

### 3.6. Caspase 3/7 Activity

**Figure 6:**
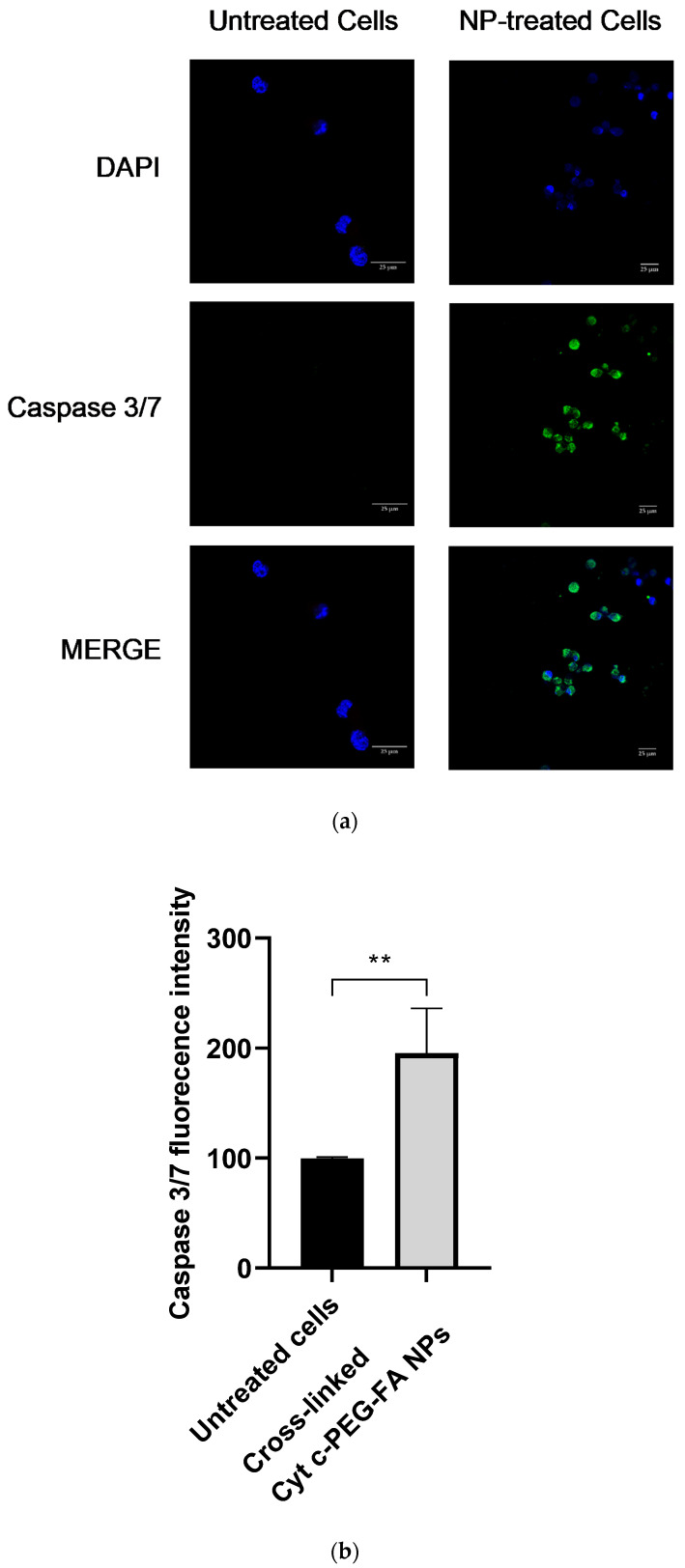
Caspase 3/7 activation in LLC cells after 24 h of incubation with 100 μg/mL of crosslinked Cyt c–PEG–FA NPs. The caspase 3/7 activity was assayed by CellEvent^TM^ caspase 3/7 fluorescent green detection reagent and measured by CLSM. (**a**) The left panel shows untreated LLC cells used as a negative control to establish the green autofluorescence background, and the right panel shows LLC cells treated with the NPs. Scale bar = 25 μm. (**b**) Quantitative analysis of green fluorescence (488 nm Ex.) in untreated versus NP-treated cells. The results are expressed as mean ± SD and were significantly different (** *p* = 0.003, unpaired *t*-test analysis, *n* = 3).

Cyt c-mediated apoptosis of a cell involves the activation of caspase 3/7. Exposure of LLC cells to NPs for 24 h presented a green fluorescence signal induced by the generated caspase 3/7 activity, while the untreated control cells showed a significantly lower fluorescence signal, as was observed by confocal imaging (Figure 6a). A quantitative analysis of these images (in the 488 nm Ex. channel shown in green) revealed a significant difference (** *p* = 0.003) between non-treated and NP-treated LLC cells (Figure 6b). These results are consistent with the cell-free caspase assay results described in Figure 2, indicating that the crosslinked Cyt c–PEG–FA NPs can substantially increase the apoptosis cell death by cellular events and molecular pathways of caspase regulation.

### 3.7. Cellular Internalization of Cross-Linked Cyt c–PEG–FA NPs

**Figure 7:**
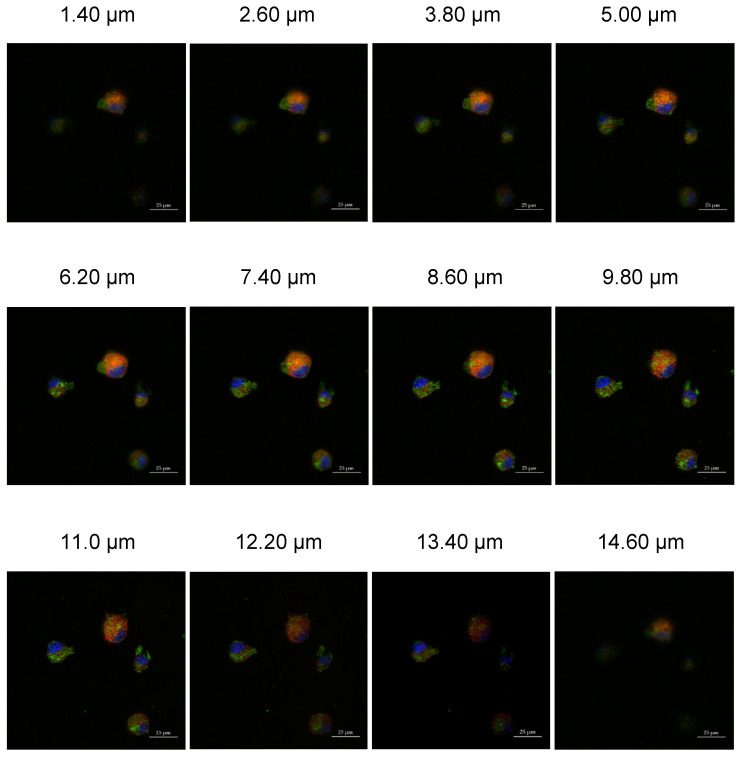
Endosomal colocalization of Cyt c–PEG–FA NPs in LLC cells using Z-stack confocal imaging. LLC cells were incubated with FITC-labeled NPs (green fluorescence) at 100 μg/mL concentration and FM-464 endosome marker (red fluorescence) for 24 h. The cell nuclei were stained with DAPI, shown in blue. The yellow color indicates the localization of the NPs in the endosomes. Scale bar = 25 μm.

To investigate the FR-mediated endocytosis mechanism and endosomal escape capability of the crosslinked Cyt c–PEG–FA NPs, FR-positive LLC cells were incubated with FITC-labeled crosslinked Cyt c–PEG–FA NPs and the endosome marker FM-464 for 24 h. The fluorescence intensity of FITC-labeled NPs in LLC cells was observed by Z-stack confocal laser scanning microscopy. The confocal Z-stack images obtained from the endosome marker channel showed red fluorescence, while the images of nuclei stained by the DAPI showed blue fluorescence. The green fluorescence observed in the confocal Z-stack images is caused by the internalization of the FITC-labeled crosslinked Cyt c–PEG–FA NPs (Figure 7). The yellow fluorescence is due to the colocalization of the two dyes and shows NP entrapment in endosomes [39]. The confocal Z-stack images reveal that FITC-labeled NPs were found in the cytoplasm after 24 h. Furthermore, some of the FITC-labeled NPs were still entrapped in endosomes (yellow spots), agreeing with the expected uptake by endocytosis. These results confirm the potential of the crosslinked Cyt c–PEG–FA NPs for tumor-targeted drug delivery.

**Figure 8:**
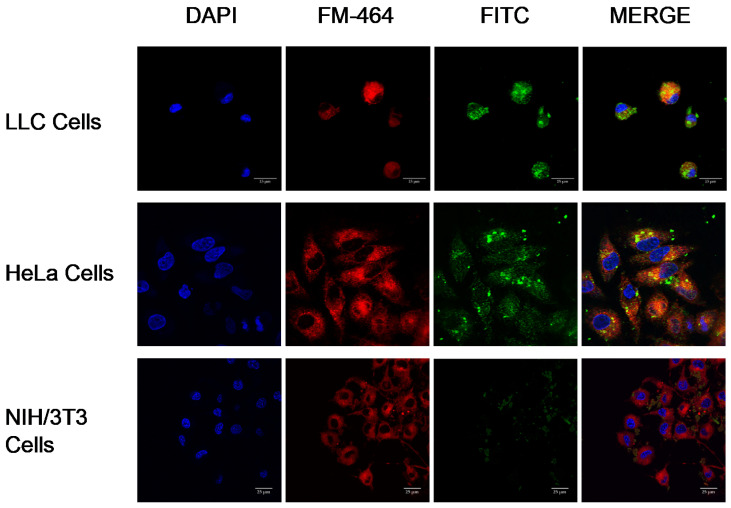
Internalization of FITC-labeled crosslinked Cyt c–PEG–FA NPs by FR-positive cancer LLC and HeLa cells and FR-negative non-cancer NIH/3T3 cells. Confocal images of both cells treated with FITC-labeled crosslinked Cyt c–PEG–FA NPs and endosome marker (FM-464) after a 24 h incubation. The yellow color in the merged images indicates the localization of the NPs in the endosomes. The nuclear stain DAPI is shown in blue. Scale bar = 25 μm.

The internalization of crosslinked Cyt c–PEG–FA NPs by non-cancer NIH/3T3 cells was also tested to confirm the specificity of our NPs to cells overexpressing folate receptors by confocal microscopy. For this purpose, FR-positive cells (LLC and Hela cells) were compared with the folate-negative NIH/3T3 cell line. Consistent with the NP uptake experiment described above, a significant amount of green fluorescence from FITC-labeled NPs was located in the cytoplasm of the FR-positive cell lines, and the colocalization with FM-464 (yellow spots) indicates NPs were also present in endosomes (Figure 8). These results indicate that the FITC-labeled NPs escaped from endosomes of the FR-positive cancer cells within 24 h of endocytosis. However, in FR-negative NIH/3T3 cells, NPs remained mainly accumulated extracellularly, and only weak yellow spots were observed in the micrographs, indicating no overlap between FITC-labeled NPs and endosome markers. These results confirm that the level of folate receptors on the cell surface affected the drug internalization. Because of the undetectable level of FR at the normal NIH/3T3 cell surface, the intracellular uptake of the crosslinked Cyt c–PEG–FA NPs decreased, and the intracellular drug release was reduced, which resulted in minimal uptake.

### 3.8. Biodistribution of Cross-Linked Cyt c–PEG–FA NPs in Tumor-Bearing Mice

**Figure 9:**
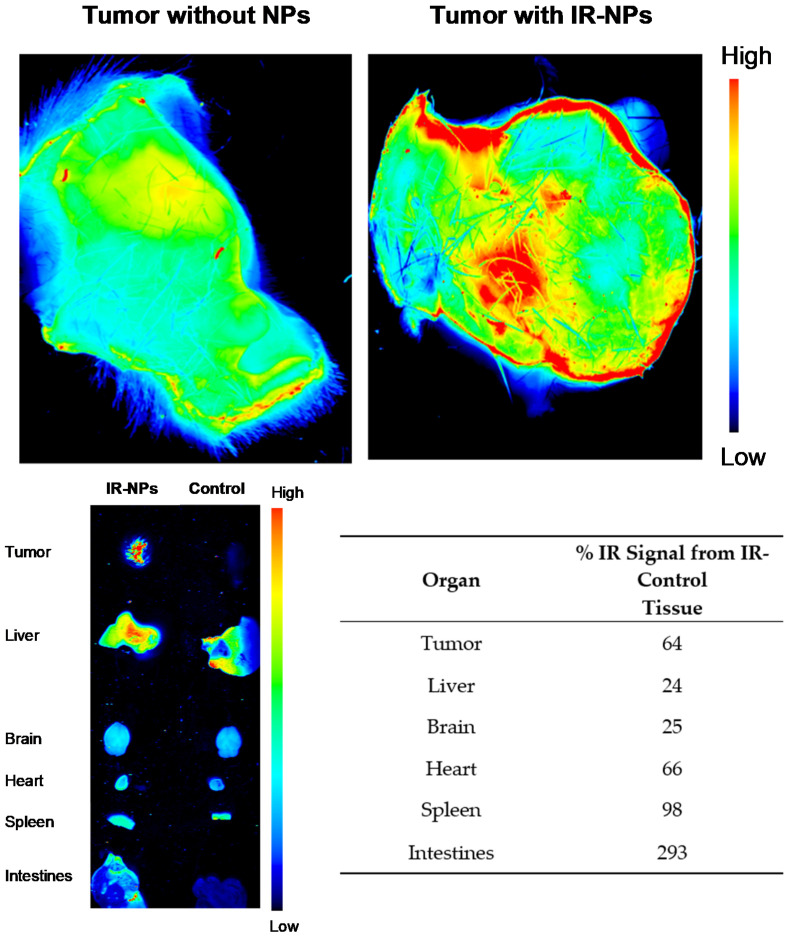
Infrared (IR) signal of organs and tumors after injection of IR-labeled-NPs. Upper panel: A high-resolution image of the ventral sides of tumors shows what appear to be NPs in the NP-injected mouse tumor but not in the control mouse. Lower-left panel: Five minutes after tail vein injection of IR-labeled nanoparticles or no nanoparticles into LLC tumor-bearing mice, tumors and organs were excised and scanned for IR signal at 680 nm (high intensity in red and low intensity in blue) using an infrared scanner (LI-COR). Lower-right panel: Percentage of IR signal in the organs of an IR-NP-injected mouse after 5 min compared to control.

For the in vivo tracking of the NP distribution, C57BL6J male mice (14-week-old) bearing Lewis lung carcinoma were tail-vein injected with 0.15 mg of NPs labeled with the fluorescent IRDye 680RD (IR-labeled NPs). After 5 min post-injection, tumors and organs (brain, heart, lungs, spleen, kidneys, liver, and intestines) were quickly extracted and scanned using the LI-COR Odyssey CLx infrared scanner. High-resolution images showed an enhanced fluorescence signal (64%) from the tumor region of the mice injected with IR-labeled NPs compared to the mice injected with PBS, which was used as a negative control to subtract the fluorescence signal from the tumors’ autofluorescence (Figure 9). A quantitative analysis of fluorescence images of the other tissues, including spleen (98%), heart (66%), and intestines (293%), showed organ deposition of a considerable amount of NPs, whereas fluorescence in the liver (24%) and brain (25%) was minimally detectable. The fluorescence signal recovered from the tumors and organs was presented as a % IR signal of NP-injected tissue over the control tissue. Therefore, our results demonstrate a successful tumor accumulation of the crosslinked Cyt c–PEG–FA NPs.

### 3.9. Induction of Lewis Lung Carcinoma in Mice to Assess NP Efficacy on Tumor Growth

**Figure 10:**
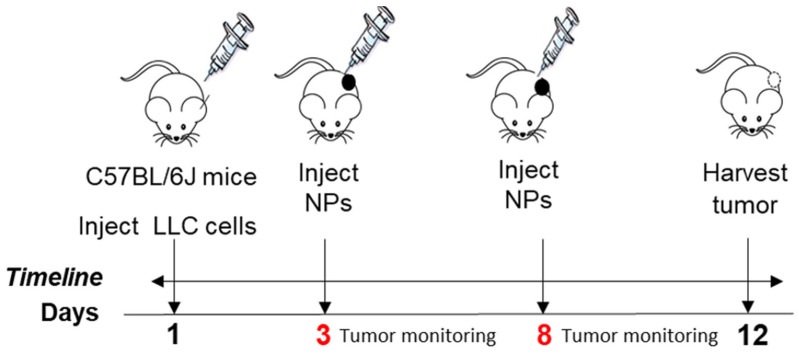
Treatment regime for C57BL/6J mice. Male mice (36–60 weeks) were divided into two groups and treated with 90% PEG 400/10% ethanol vehicle (i.p.) or 7 mg/kg of NPs dissolved in a vehicle (i.p.) at days 3 and 8 after the tumor implant. Tumor monitoring was performed manually by caliper measurement at days 3, 6, 9, and 12, and weight was monitored as a general health measurement in our mouse model.

**Figure 11:**
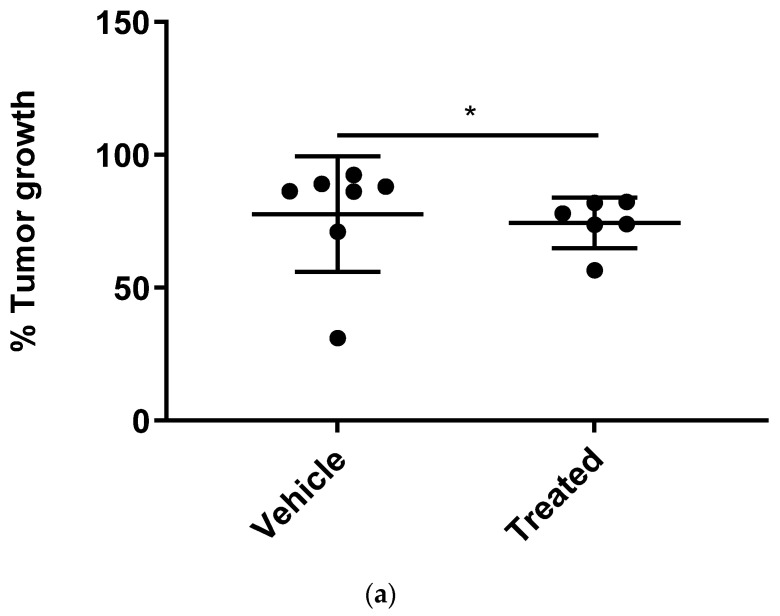

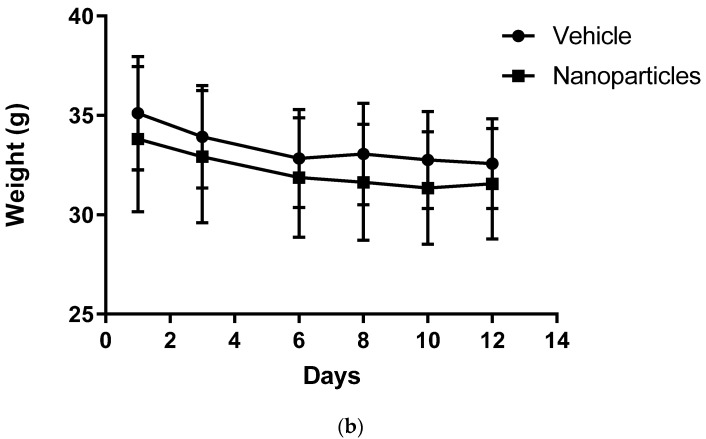
Percentage of tumor growth and mouse weight in NP-treated and untreated mice. (**a**) Tumor growth monitoring was performed in male mice manually by caliper measurement. (**b**) Mouse weight was monitored using a rodent balance. A *t*-test and Kolmogorov–Smirnov post-test on percentage tumor growth showed * *p* = 0.0385, while mouse weight was not significantly different between both groups. Vehicle (*n* = 6) and NP-treated (*n* = 7).

C57BL/6J mice were implanted with LLC cells to each grow a tumor for a total of 12 days. For the NP treatment, mice were injected intraperitoneally with 7 mg/kg of crosslinked Cyt c–PEG–FA NPs (169 nm) at day 3 as an early-stage tumor intervention, and at day 8, as a late-stage intervention in tumor growth. Figure 10 shows a representative image of the regime used. Tumor volume was measured manually by caliper every 3 days using the length and width of tumors, and the mice’s weight was also monitored as a general health measurement. Using an unpaired *t*-test with a Kolmogorov–Smirnov post-test between the NP-treated and untreated mice showed a significant decrease in tumor size in NP-treated mice (*p* = 0.0385, Figure 11a).

Our results show no significant difference in mouse weight between groups (Figure 11b). This underscores the safety and suitability of the crosslinked Cyt c–PEG–FA NPs as a DDS for FR-overexpressing tumor therapy. These results are consistent with the in vitro therapeutic efficiency and the in vivo tumor accumulation of the crosslinked Cyt c–PEG–FA NPs.

## 4. Discussions

Considering the many advantages of protein-based NPs to facilitate their clinical applications and the exemplary success of anticancer NP-based drug formulations, such as Abraxane^®^, our results show a proof-of-concept that crosslinked Cyt c–PEG–FA NPs have the potential to improve tumor-targeting and anti-tumor effects in drug delivery. Our fast infrared NP detection system could be used as a tumor diagnostic agent for folate overexpressing cancers, and further development of the system could have theragnostic potential [40]. Previously, Cyt c NPs stabilized by the hydrophobic polymer poly (lactic-co-glycolic) acid (PLGA) demonstrated that these NPs were an efficient method to induce cell death in lung carcinoma cell culture, and bound to the tumor site in the LLC murine model [29]. Recently, we overcame dose limitations previously seen in the PLGA-based NPs for Cyt c by designing smaller Cyt c-based NPs coated with a low-molecular-weight polymer, FA–PEG. In the current study, Cyt c NPs were stabilized using a thiol-cleavable homo-bifunctional crosslinker that incorporates a triggered release mechanism mediated by the reducing environment inside the cancer cells—without the need for PLGA. This NP formulation showed improved cytotoxicity and biocompatibility compared to a previous report [25]. In addition to having an optimal and reduced NP size (~169 nm, compared to the previous system of ~254 nm), this new generation of NPs is more straightforward and economical to prepare, which is critical to the development of accessible anticancer drugs. Reviews of NPs have concluded that after many studies of the sizes, shapes, and surface modifications of NPs, a suitable size for NPs targeting tumors is 100–200 nm. These particles display increased tumor penetration, because they are large enough to avoid being cleared by the kidneys or through vascular extravasation (which eliminates particles of 10–100 nm) [41,42], and they are still large enough to avoid filtration by the kidneys and spleen (300–500 nm) [23]. Our presented NPs of 169 nm fall within this optimal size. This should also improve their passive tumor entry and accumulation due to the enhanced permeability and retention effect (EPR) caused by the unstable tumor vascularization, which leads to a better drug efficacy within the tumor microenvironment [26].

Studies have shown that increasing the flow rate of the solvent and antisolvent during the nanoprecipitation process can cause a considerable reduction in the particle size in nanosuspension [43]. Therefore, in these studies, the nanoprecipitation method was optimized by increasing the flow rate of the solvent-displacement process two-fold, resulting in a diameter of 169 nm—a 30% reduction compared to the previous system—and producing a positive NP surface charge (+17 mV). For this system, our strategy was to modify the surface of the Cyt c NP with both a redox-responsive homo-bifunctional crosslinker (DSP) and a lower-molecular-weight polymer (FA–PEG), making it a potential candidate for intravenous (i.v.) administration [3,26]. Whereas the crosslinker shell stabilized the core of the Cyt c-based NP, the polymer provides the surface for FR-overexpressing tumor targeting. The SEM images of the crosslinked Cyt c–PEG–FA NPs showed a spherical shape with narrow size distribution, and confirmed the nanometer range of the NP size determined by DLS. Previous limitations of protein delivery systems, including low protein loading and poor protein stability, also improved with the crosslinker.

The DSP is a cell-membrane-permeable crosslinker with a disulfide bond that can be reduced in the intracellular environment, preventing the disintegration of the NP in an aqueous environment [44]. Our in vitro drug release results show an excellent release of around 90% of Cyt c in 2.5 h under reducing intracellular conditions and high stability under extracellular physiological conditions. However, protein structures can respond to changes in their chemical and physical environment in the NP formulation process [45]. Cyt c’s action as an anticancer drug depends on the mitochondrial apoptosis pathway, which is responsible for activating the executioner caspases 3/7 that target various protein substrates, leading to cell disassembly and DNA fragmentation [46]. We demonstrated that the crosslinked Cyt c–PEG–FA NPs retained all their enzyme activity (94 ± 8%) through a cell-free caspase 3/7 activation assay, meaning that the integrity of Cyt c after the NP-formulation procedure was not compromised. This result is significant, because many of the Cyt c surface lysine residues are known to be involved in the Apaf-1 interaction, which is essential to the mitochondrial apoptosis pathway [47]. Therefore, we demonstrated that our delivery system displayed high stability in physiological conditions and smart-release behavior in a reductive intracellular environment, retaining all its protein bioactivity to interact with the Apaf-1 and activate the apoptosis pathway.

Conventional chemotherapy limitations arise from a lack of specificity and systemic toxicity without the discrimination of healthy tissues. Therefore, a cell viability study was conducted to investigate the cytotoxic potential of the crosslinked Cyt c–PEG–FA NPs in lung carcinoma cancer cells. We used the Lewis lung carcinoma (LLC) cell line as the lung carcinoma model because it expresses high levels of FRs, is highly tumorigenic, and is primarily used to evaluate the efficacy of chemotherapeutic agents in vivo [48]. For example, the LLC mouse model was a successful in vivo preclinical prototype for assessing the drug called Navelbine^®^ before human testing in clinical trials [49]. Our in vitro cell viability studies demonstrate that the crosslinked Cyt c–PEG–FA NPs were significantly more cytotoxic towards FR-overexpressing cancer cell lines, including human HeLa cancer cells. In contrast, no significant or minimal cytotoxicity was observed in the non-cancerous NIH/3T3 cell line. In addition, HeLa cell death induced by the NPs demonstrated the translational application of our system to human FR-overexpressing cancers. Thus, the selective cytotoxic efficacy of the crosslinked Cyt c–PEG–FA NPs in FR-receptor-overexpressing cancer cells over an FR-negative non-cancer cell line was confirmed.

The cellular apoptotic process induced by Cyt c is one of two apoptotic mechanisms, named ‘intrinsic’, ‘mitochondrial’, or ‘stress-induced’. In this intrinsic pathway, a sequential protein activation process leads to the release of Cyt c from the mitochondria, which in turn activates caspases 9, 3, and 7, which mediate the mechanisms of organized cellular death [50]. Among these processes, we can observe apoptotic cells displaying their characteristic nuclear segmentation and chromatin condensation [51]. For visualization of apoptosis, DAPI and PI colocalization studies have been used to detect apoptotic cells [52]. Our results showed that colocalization of DAPI and PI occurred in LLC cells, indicating ongoing late apoptosis, whereas NIH/3T3 cells showed no indication of dye colocalization. These results demonstrate that the crosslinked Cyt c–PEG–FA NPs induced selective cell death in FR-overexpressing cancer cells without affecting healthy cells, thus confirming these NPs’ selectivity regarding cancer cells.

To expand the molecular and cellular mechanism underlying the early apoptotic induction of the NP treatment in LLC cells, we examined fluorescent caspase 3/7 activation in cell cultures using confocal microscopy and a cell-free assay. Both methodologies confirmed that the Cyt c protein carried by our targeted-PEGylated NPs effectively initiated tumor cell apoptosis in cancer cells.

Because our NPs were smaller than 500 nm and had an FA ligand attached to their surfaces, they could be internalized through receptor-mediated clathrin-enabled endocytosis [53]. This internalization mechanism is initiated after a specific ligand-encased nanomaterial binds to a receptor on the surface of the cell membrane. After 24 h of LLC cell incubation with our FITC-labeled crosslinked Cyt c–PEG–FA NPs, the amphiphilic dye FM 464 was used as our endosome marker to confirm the uptake of our NPs in FR-positive cells. Our Z-stack confocal microscopy results showed a colocalization of FM 464, with our fluorescently labeled NPs showing a successful uptake.

Further confocal micrograph studies showed a significantly higher intracellular uptake of PEG-FA NPs by FR-positive cancer cells (HeLa cells and LLC) over FR-negative cells (NIH/3T3), indicating that the internalization of these NPs was specific and mediated via an FR-receptor and clathrin-mediated endocytosis mechanism. The internalized FITC-labeled NPs were identified inside the cytoplasm and surrounding the nucleus in the FR-overexpressing cell lines. Overall, our endocytosis results suggest that our NPs had an appropriate size, shape, adequate surface charge, and coating to be efficiently internalized by cancerous cells, and could have potential as anticancer nanocarriers [54].

Our in vivo studies demonstrated tumor targeting by showing that, at 5 min after intravenous injection, the NPs were visible within the tumor. At this early time point, most NPs were still present within organs including the intestines, spleen, heart, brain, and liver. From our experience with similar NPs, these would be later metabolized after 6 h [29]. Nevertheless, further toxicological analyses of the organs at different time points after NP treatment need to be performed to confirm the elimination of this drug.

Our studies determined that the crosslinked Cyt c–PEG–FA NPs were able to reach the tumor, and we further tested the safety and tumor decrease effectivity of these NPs in vivo. Results show that after two doses of NPs at 7 mg/kg, after 12 days, mice showed no significant differences in weight compared with the vehicle group (control); they looked groomed, changes in locomotion were not detected, and they showed no evident signs of toxicity throughout the treatment period. These results suggest that the NPs are safe and have low toxicity, which could make their use for future clinical translation possible. In addition to their safety, our NPs also significantly decreased the percentage of tumor growth consistently by at least 5%. Future improvement of these NPs’ formulation, dosage, and combined therapy could increase the treatment’s outcome, but it already shows promising results as the first in vivo proof-of-concept trial. Other FR-targeted DDS loaded with well-known anticancer drugs have shown positive results, decreasing tumor growth in vivo [55,56] and reducing their toxic effects by nanoencapsulation. Our approach serves as a platform for the creation of drug delivery systems employing apoptosis-inducing or other pharmaceutical proteins for various therapeutic applications.

## 5. Conclusions

In the present work, we report the development of a crosslinked Cyt c–PEG–FA NP drug delivery system that was designed for FR-mediated targeting and the intracellular redox-sensitive release of the apoptotic protein Cyt c. Our results indicate that our NPs had a significant tumor-growth suppressive effect on cancer cells with FR overexpression in vitro and in vivo. The in vitro results demonstrate that our NPs were adequately sized for tumor targeting, selectively internalized in FR-overexpressing cancer cells, and able to induce apoptosis, activating a caspase 3/7 mechanism without affecting normal cells. Indeed, our results strongly suggest that the size of an NP plays an important role in the cytotoxic effect compared with our previous Cyt c-based delivery system stabilized by PLGA. Our in vivo studies using an immune-competent mouse model of lung carcinoma demonstrated tumor targeting by showing that at 5 min after intravenous injection, the NPs were visible within the tumor. In the same mouse model, our NPs effectively suppressed tumor growth, most probably through the apoptotic activation of caspase 3/7 in tumor cells, without notable side effects. Therefore, these results showcase the potential of our crosslinked Cyt c-based NPs for targeted anticancer therapeutics, avoiding problems associated with many of the common cytotoxic anticancer agents, such as their unspecific targeting of healthy cells and drug resistance.

## Figures and Tables

**Table 1:** Size, polydispersity index, zeta potential, and precipitation efficiency of different NPs prepared by the nanoprecipitation method.

NPs	Diameter (nm)	Polydispersity Index (PDI)	Zeta Potential (mV)	Precipitation Efficiency (%)	Actual Loading (%)
Cyt c–DSP NPs	164 ± 5	0.06 ± 0.01	27.5 ± 3.9	96 ± 2	80 ± 3
Cross-linked Cyt c–PEG–FA NPs	169 ± 9	0.09 ± 0.01	17.7 ± 1.7	97 ± 3	74 ± 6

Table data show the averages of three batches of NPs prepared and the respective standard deviations.

## Data Availability

Not applicable.
